# Giant caldera in the Arctic Ocean: Evidence of the catastrophic eruptive event

**DOI:** 10.1038/srep46248

**Published:** 2017-04-10

**Authors:** Alexey Piskarev, Daria Elkina

**Affiliations:** 1 Institute of Earth Sciences, St. Petersburg State University, 7/9 Universitetskaya emb., St. Petersburg, 199034, Russian Federation; 2 All-Russian Research Institute of Geology and Mineral Resources of the World Ocean (VNIIOkeangeologia), 1 Angliysky Avenue, Saint-Petersburg, 190121, Russian Federation

## Abstract

A giant caldera located in the eastern segment of the Gakkel Ridge could be firstly seen on the bathymetric map of the Arctic Ocean published in 1999. In 2014, seismic and multibeam echosounding data were acquired at the location. The caldera is 80 km long, 40 km wide and 1.2 km deep. The total volume of ejected volcanic material is estimated as no less than 3000 km^3^ placing it into the same category with the largest Quaternary calderas (Yellowstone and Toba). Time of the eruption is estimated as ~1.1 Ma. Thin layers of the volcanic material related to the eruption had been identified in sedimentary cores located about 1000 km away from the Gakkel Ridge. The Gakkel Ridge Caldera is the single example of a supervolcano in the rift zone of the Mid-Oceanic Ridge System.

While the mankind looks deeper and deeper into the space over the past decades, detailed study of the bottom topography of the Arctic Ocean has started quite recently. Until the middle of the last century, there were a few sporadic studies of the deep-water part of Arctic Ocean. Based on the eleven depth measurements taken by F. Nansen during his famous “Fram” voyage at the end of XIX century, it was for a long time considered as vast undifferentiated depression. A new phase of the Arctic Ocean investigations began in the late 40 s of the last century with the Soviet high-latitude airborne expeditions under the leadership of J.J.Gakkel. By 1956, the Soviet polar explorers measured depths at more than 400 locations, discovered the Lomonosov Ridge and published the bathymetric maps which radically changed the image of the Ocean floor topography^1^,^2^,^3^,^4^.

In the second half of the last century, the American explorers discovered the Alpha Ridge in the central part of Arctic Ocean^5^,^6^. The US Navy explored the Arctic Basin using nuclear submarines: *Nautilus, Skate*, and *Sargo*^5^,^6^,^7^.

By the end of 60 s decade, up to 20,000 depth measurements by expeditions from different countries were accumulated and, despite their uneven distribution, the basic oceanographic units of the Arctic Ocean were established: the Eurasian and Amerasian basins separated by the Lomonosov Ridge, Nansen and Amundsen abyssal basins, separated by the Gakkel Ridge (northern continuation of the Mid-Atlantic Ridge)^2^,^5^. In the 1970s, the southern end of the Gakkel Ridge was extended on the bathymetric maps to the edge of Laptev Sea continental margin up to 79°30′N. However, due to lack of data, topography of the Ridge itself was represented in idealized form as an ordered system of more than 25 parallel transform faults constantly changing the direction of the axial rift valley, reflecting subjective perception of similarity between known mid-ocean ridges.

The later studies have significantly expanded bathymetric database of the abyssal Arctic Ocean and helped to provide detail representation of underwater topography.

## Methods

### Bathymetry data

The morphology of the present-day Arctic Basin was imaged from the available bathymetry data: IBCAO digital grid, v. 3.0^8^, uploaded and processed in the Geocap software^9^ for visualization.

### Acoustic soundings

The Marine Arctic Geological Expedition (MAGE) performed seabed mapping together with seismic surveying onboard R/V *Akademik Fyodorov*. The survey was carried out in ice, and R/V followed after nuclear-powered icebreaker *Yamal*. An EM-122 multibeam echosounder, Kongsberg Maritime was applied for seabed mapping with swath width of about 30 km. In addition, an EA-600 single-beam echosounder, Kongsberg Maritime was used simultaneously.

### Seismic survey

2D multichannel reflection seismic survey accompanied with multibeam bathymetry was carried out by MAGE onboard R/V *Akademik Fyodorov*. Recordings were performed with Sercel SEAL System, ver.5.1. The seismic streamer was 4500 m long with Geopoint Export hydrophones installed. The most seismic lines had a source tow depth of 15 m. Pickets were separated by a distance of 50 m, record time was 12 s. There were 8 seismic source points of APG BOLT-8500 type, and an active volume was 1300 cubic inches (21 l).

### Paleomagnetic data

**Figure 1:**
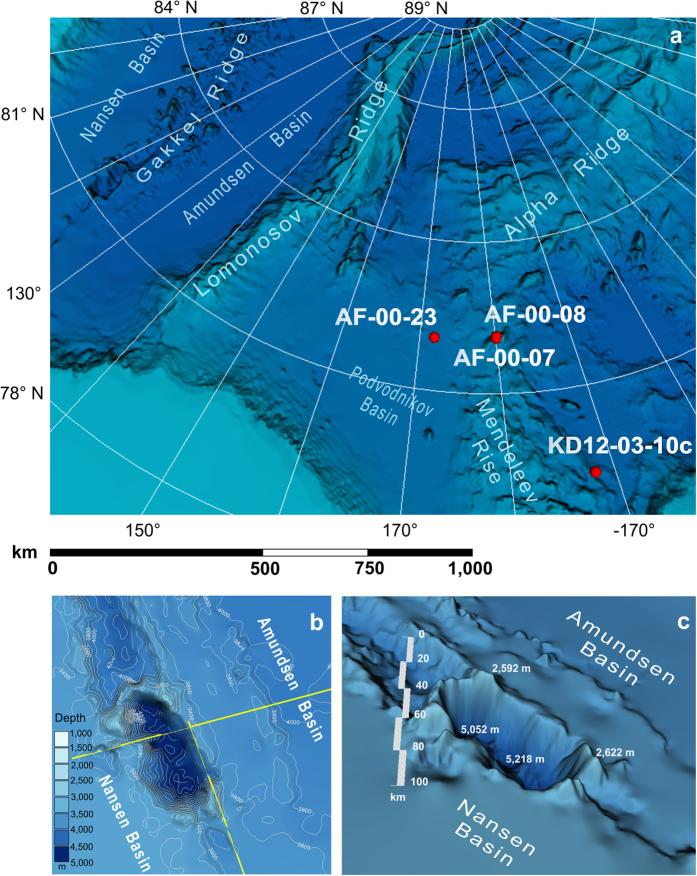
(**a**) View of the Arctic Basin structures and coring sampling sites; (**b**) caldera on the Gakkel Ridge rift valley (yellow lines - MCS seismic lines and multibeam survey, 2014); (**c**) 3D view of caldera and surrounding ocean floor topography (Geocap software^9^ on IBCAO grid v. 3.0^8^).

For paleomagnetic studies, specimens were sub-sampled from undeformed sediment cores recovered on the Mendeleev Rise area in 2000^10^ and 2012^11^ (Fig. 1a). Specimens were obtained by pressing glass cylinders of 23 × 23 mm size in average into the sediments. Thus, the cylinder size determines the resolution of measurements. All the cylinders were oriented relative to the core coordinates.

The specimens of 2000 were studied for natural remanent magnetization (NRM) and magnetic susceptibility (***k***). The ***k*** measurements were carried out using a KLY-2c magnetic susceptibility meter with a sensitivity of up to 4 × 10–8 SI and a calibration accuracy of ±3%. The NRM intensity and direction were measured using a JR-4 spinner magnetometer, AGICO with a vector component measurement accuracy of ±1% and with a vector direction error of no more than 10°.

For the 2012 set, magnetic (volume) susceptibility of 1st and 2nd meters of the 6-meters long core was obtained from discrete measurements of specimens by a Kappabridge MFK1-FA, AGICO. Starting from the 3rd meter, magnetic susceptibility was measured by Kappameter KT-5 and MS2E surface sensor, Bartington on the non-disturbed core surface with 6.5 and 2.5 cm intervals respectively. Measurements of NRM were carried out with a spinner magnetometer JR-6A, AGICO. Demagnetization procedures were performed for the specimens of interest, selected by analysis of data, acquired from the NRM measurements. The specimens, treated by temperature, were heated at 130 °C and further by 50 °C steps in 200–450, 500 °C range using a thermal specimen demagnetizer TD-48-SC, ASC Scientific. The alternating field (AF) demagnetization was done with a 2G-Enterprises SRM-755 cryogenic magnetometer at 16 steps in the 5–160 mT peak field range. Characteristic remanent magnetization, used as a main characteristic for the final magnetostratigraphy, was determined mostly in the 250 °C–450 °C heating interval for thermally demagnetized specimens and in over the range of 20–100 mT for AF demagnetized ones using for both cases the principle component analysis method^12^. The paleomagnetic measurements on core KD12-03-10C were performed at the Research park of St. Petersburg State University Center for Geo-Environmental Research and Modeling (GEOMODEL).

All the studied sediment cores were retrieved close to the North Pole and the most important paleomagnetic parameter at such high latitudes is an inclination of the geomagnetic field. Declination information was not considered due to an arbitrary orientation of the core in the horizontal plane.

### Mineralogical analysis

Mineralogical analysis of light and heavy suites was performed on 0.1–0.05 mm fraction after its separation by heavy liquid (bromoform). Based on optical property sets, minerals were identified using an Olimpus BH-2 bin-ocular microscope

### Thin sections

Dry oriented specimens obtained during the paleomagnetic study mentioned above were boiled in Canada balsam in order to fix their initial structure and mode of arrangement. Then thin sections were prepared. Petrological description of the latter contained rock type and rock family, mineral assemblage, grain size, habitus, unique features, and, moreover, composition, structure and mode of arrangement of the bulk of a thin section.

## Giant Caldera

The caldera was identified for the first time on the International Bathymetric Chart of the Arctic Ocean (IBCAO), v. 1 (2.5 × 2.5 km grid) compiled by group of international experts and presented to the American Geophysical Union in 1999. Figure 1b and c shows the fragments of the IBCAO latest version with the caldera clearly visible.

The caldera is located astride and along the axis of the Gakkel Ridge rift valley with the center at 81°31′N, 120°00′E, where the Gakkel Ridge topography gradually flattens out approaching the edge of Laptev Sea shelf. The caldera is 40 km wide, 80 km long and 1.2 km deep; numerous peaks, mounds and ridges up to 0.5–1 km high are visible along its rim. The expected total volume of volcanic material ejected during formation of the caldera is at least 3000 km^3^ as one can calculate from caldera’s depth and size. This puts it at par with the largest Quaternary calderas on Earth: the Yellowstone and Toba.

**Figure 2:**
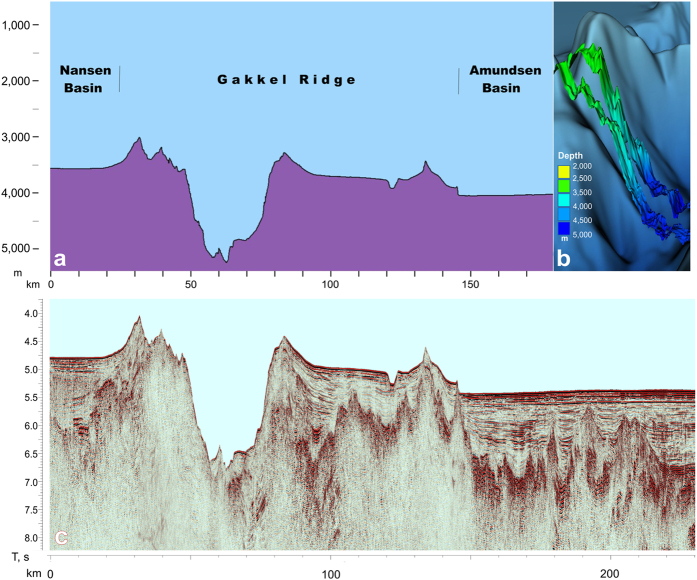
(**a**) Cross section of the caldera according to multibeam echosounding, line 2014-05; (**b**) View at the western slope of the caldera with multibeam echosounding lines superimposed on IBCAO grid v. 3.0^8^ (Geocap^9^); (**c**) seismic section, line 2014-05.

Seismic and multibeam echosounding data acquired in 2014 (see Fig. 1b for profiles location) brought to light the important information on recent tectonic history of the Gakkel Ridge and Eurasian Basin. The data highlight the details of caldera slopes and demonstrate that the contemporary tectonically active rift valley (divergent plate boundary) 10 km wide and 500 m deep is visible at the caldera floor 4800 m below sea level (Fig. 2).

The first discernible seismic reflections from sedimentary layers appear on both sides of caldera 20–30 km away from caldera walls pointing to massive magmatic composition of the rim. The maximum thickness of sediments on the ridge flanks is estimated at 1 km (approximately 1 second 2WT). Several steps on the lower part of both wall seems to demonstrate that their fine details were sculptured by normal faulting.

Assuming average rate of spreading in the Eurasian Basin about 1 cm per year^13^, it took approximately 1 million years to form the 10 km-wide rift valley at the bottom of the caldera. Therefore, the very important stage of tectonic evolution of the Eurasian Basin– intensive magmatic activity and creation of the giant caldera – came to a close at around 1 million year ago.

## Evidence of eruption traces in the sediments

Introduction of a huge amount of volcanic material in the waters of the Arctic Ocean should have left a noticeable impact on composition and properties of sediments formed during and after the eruption. Therefore, wide distribution of omnipresent sedimentary layers of volcanogenic origin is expected. Such layers can be identified by increased concentration of monoclinic pyroxenes and opaque ore minerals^14^,^15^,^16^, and by higher values of magnetic susceptibility and residual magnetization caused by higher concentration of magnetite and titanomagnetite.

**Figure 3:**
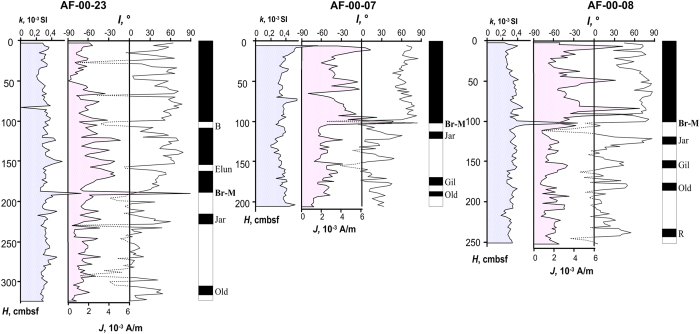
Paleomagnetic correlation of sedimentary cores collected during cruise of R/V *Akademik Fedorov* (2000). (1) Magnetic susceptibility, ***k***; (2) Natural Remanent Magnetization, ***J***; (3) angle of inclination, ***I***; (4) zones of normal (black) and reverse (white) polarity of the geomagnetic field. See Fig. 1a for location.

Several such layers, with anomalously high values of magnetization and magnetic susceptibility were identified in sedimentary cores collected in the Mendeleev Rise, about 1000 km away from the caldera (Fig. 1a), and dozens of kilometers from each other. One of them stratigraphically is very close to the Brunhes/Matuyama chron and could be dated with high degree of confidence at about 750 Ka (Fig. 3)^10^. This layer, only a few centimeters thick, is clearly correlatable from one core to another despite the fact the cores are separated by a considerable distance.

**Figure 4:**
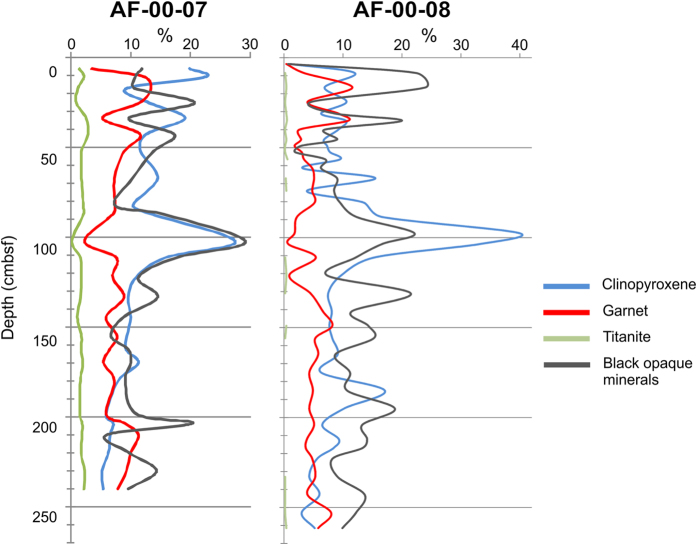
Mineralogical analysis of heavy fraction (cores AF-00-07 and AF-00-08, Mendeleev Rise).

The cores mineralogical analysis demonstrates sharp increase of pyroxene and ore minerals and depletion of garnet and titanite (indicators of “stable” depositional environment) in 750 Ka layer (Fig. 4).

Increased concentrations of ore minerals and pyroxene indicate a probable volcanogenic nature of the studied thin sedimentary layer. At the same time, a significant decrease in concentration of obviously clastic minerals as garnet and titanite means an abrupt surge of the sedimentation rate during the formation of this volcanogenic layer in the sedimentary column. Apparently, for a short period of time there was five times increase of sedimentation rate comparing to its average value.

**Figure 5:**
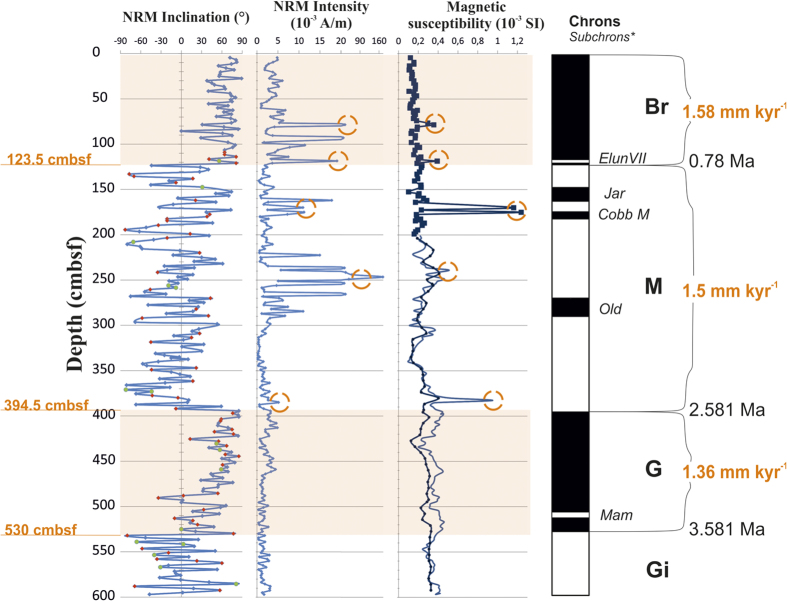
Paleomagnetic correlation with the Geomagnetic Polarity Time Scale^24^,^25^ on sedimentary core KD12-03-10C. The mean sedimentation rates have been determined for each paleomagnetic chron based on the established paleomagnetic boundaries, and related age^26^,^27^. Yellow circles mark abnormally high values of the magnetic susceptibility, and natural remanent magnetization (NRM). See Fig. 1a for location.

Even more valuable information on the specifics of the Pliocene-Quaternary sedimentation was obtained from core KD12-03-10C extracted on the Mendeleev Rise in 2012 (see Fig. 1a for location)^11^. The 6 m-long core represents the time interval down to ≈4 Ma (Gilbert paleomagnetic chron) - Fig. 5.

Distribution of NRM inclinations with depth displays alternating intervals of normal and reverse polarity of paleogeomagnetic field. Starting from the top of the core, the positive inclination prevailed up to 123.5 cmbsf (cm below sea floor) when changed sharply to the negative which, with some short-lived events of normal polarity, remain prevalent up to 394–397 cmbsf. On average, magnetization of the intervals with the positive inclination is stronger than those with negative one. This difference can be explained by the presence of viscous component of the magnetization, which was also noted in several other previously collected columns.

Visually, the bulk of core KD12-03-10C is relatively homogeneous aleuropelite (silty clay). However, measurements of magnetic properties (magnetization and magnetic susceptibility) performed at fine intervals (2.5 cm) have identified five horizons with peak values of magnetization and magnetic susceptibility, which are marked by yellow circles on Fig. 5. Those five identified intervals were then dated by correlation with the paleomagnetic chrons:

77.5 cmbsf (0.47 Ma),

118.5 cmbsf (0.727 Ma),

170–175 cmbsf (1.09 Ma),

240 cmbsf (1.62 Ma),

385 cmbsf (2.52 Ma).

Weight percentage distribution of the coarse fraction (>500 microns) along the core demonstrated correlation between intervals with high coarse fraction content and the spikes of magnetic susceptibility values.

In order to identify the lithological differences between the bulk of the core and above mentioned anomalous intervals, several thin sections of both were prepared and studied.

**Figure 6:**
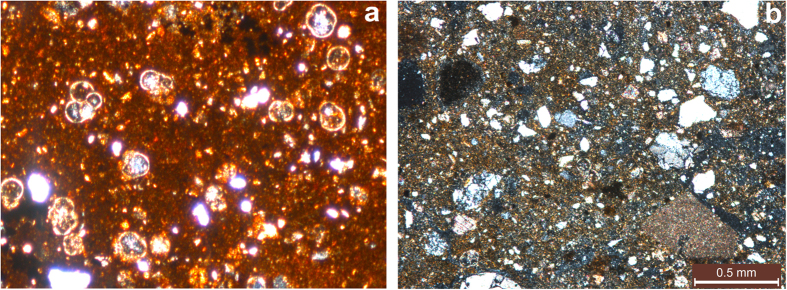
Polarizing microscope. Nicoli X. Vertical thin sections: (**a**) Horizon 114 cmbsf, bulk of the core; (**b**) Anomalous horizon 175 cmbsf, high magnetization values.

Thin section on Fig. 6a presents the bulk of the core as biogenic sediment with heavily ferruginous clay-carbonate mass, saturated by fragments of quartz, plagioclase, carbonates and planktonic foraminifera. Iron hydroxides tint the clay-carbonate basic mass in yellow-brown colors and form a small spotted brown impregnation in cement and small halos around of some foraminifera. Sediments of the marked anomalous intervals look differently, as illustrated by thin section of material at 175 cmbsf (Fig. 6b). Clastic fraction is completely unsorted neither by size, nor by degree of roundness; the color of this horizon is noticeably lighter and in general it has an appearance of tuffite; fauna fragments are remarkably rare, curved tephra shards are present, and, besides, there are noticeably more angular tephra shards up to 0.5 mm in size than in the bulk of the core. The following fragments are also typical for these horizons: hornblende, ore minerals, iron hydroxides, glauconite, calcite, and, the most importantly, higher content of clinopyroxene.

We can conclude with high degree of confidence that lithological, mineralogical, petrograhic, granulometric and geophysical properties of the described horizons demonstrate their volcanogenic origin and explosive character of volcanic activity. The detail analysis of core KD12-03-10c showed that the most pronounced episode took place 1.09 Ma ago (Fig. 5). This is very close to the time of formation of the caldera (≈1 Ма) derived from the rift valley width at the caldera floor and average spreading rate of the Gakkel Ridge.

The explosion episode and volcanic activity that led to the formation of the caldera may be a key to explain why the Gakkel Ridge rift valley in the eastern part of the Eurasian Basin is located in the south-western flank of the Ridge. This is clearly seen on the modern topography map of the Arctic Ocean (Fig. 1), and indicates a recent jump of spreading axis.

## Unique Gakkel Caldera

It could be safe to state that the Eurasian Basin of the Arctic Ocean in the Pleistocene was a scene for a unique and powerful volcanic eruption with huge volumes of ejected material. Apparently, this eruption dated at ~1.1 Ma was not a single powerful eruption in the Arctic Basin during the Pliocene and Pleistocene.

The presence of large volcanic structures was found earlier in the western part of the Gakkel Ridge during the expedition AMORE 2001^17^,^18^. Ultraslow-spreading ridges have a unique feature - amagmatic rifts that expose mantle peridotite (with only traces of basalt and gabbro) directly on the seafloor thus forming a new (fourth) type of plate boundaries^19^. These discoveries raise new questions about violent processes in ultra-slow-spreading systems^20^. We consider the described caldera as an evidence of some new form of volcanism related to this type of plate boundary. Probably it was the most powerful volcanic eruption which left the significant marks on the topography and sedimentation within the Arctic Ocean. It also might affect the recent (Pleistocene) spreading geometry of the eastern part of the Eurasian Basin by triggering a jump of the Gakkel Ridge spreading axis which, instead of typical central position, is located in its south-western flank (Fig. 1).

The size of caldera, which we suggest to call “Gakkel Caldera” due to its location, put it in category of supervolcanoes with Volcanic Explosive Index 8. This category includes volcanoes with eruptive release of more than 1000 km^3^ of lava and volcanic ash. In this case, the latter contaminated not only atmosphere but hydrosphere as well, as proven by presence of volcanic material in sediments thousands of kilometers away from the volcanic sources. The scope and duration of climatic changes related to the catastrophic Pleistocene volcanic events and their impact on all living organisms including humans are still under discussion, as a debate on ecological impact of the Toba eruption 75 Ka may attest^21^.

The tectonic position of Gakkel Caldera is a remarkably interesting feature. As far as we know, Gakkel Caldera is a unique example of a supervolcanoе formed in the rift zone of a mid-ocean ridge. All other known supervolcanoes are located above subduction zones or in the immediate vicinity as that creates the favorable conditions for generating giant magma chambers. It should be noted that, in fact, Gakkel Caldera is located at the termination of the mid-ocean ridge. Its rift valley turns to a graben of several hundred m depth verging towards the Laptev Sea shelf. Faults forming the graben’s walls are traceable deep into the thick sedimentary sequence, and, according to the data^22^, the Cretaceous sediments constitute the lower layers.

The existence of the suture zone, cross-linking the Cenozoic and Mesozoic ocean floor in this part of the Eurasian Basin, was suggested earlier^23^. However, geological and geophysical data do not allow defining precisely the nature of the zone so far. Evidently, Gakkel Caldera appears to be one of its characteristic features.

## Additional Information

**How to cite this article:** Piskarev, A. and Elkina, D. Giant caldera in the Arctic Ocean: Evidence of the catastrophic eruptive event. *Sci. Rep.* **7**, 46248; doi: 10.1038/srep46248 (2017).

**Publisher's note:** Springer Nature remains neutral with regard to jurisdictional claims in published maps and institutional affiliations.

## References

[b1] Gakkel J. J. Science and exploration of the Arctic. 133. L.: Marine transport, 1957).

[b2] Gakkel J. J. International geophysical year in the Arctic. J. Science and Life. 1, 23–26 (1959).

[b3] Gakkel J. J., Belov N. A., Dibner V. D. & Lapina N. N. Morphostructure and bottom sediments of the Arctic Basin. Tr. AARI. 285, 15–27 (1968).

[b4] Kiselev Yu. G. The structure of the seabed and the evolution of the geological structure of the deep-water part of the Arctic Ocean. Geophysical research methods of the oceans. 5–13 (Research Institute of Arctic Geology, 1979).

[b5] Ostenso N. A. Geophysical investigations of the Arctic Ocean basin. *Research Report*. University of Wisconsin. Geophysical and Polar Research Center. 4, 124 (1962).

[b6] Weber J. R. CESAR 83: Alpha Ridge probe will increase understanding of Arctic. Resource Development, Winter 1982/83. 10–12 (1983).

[b7] Dietz R. S. & Shumway G. Arctic Basin geomorphology. J. Geological Society of America Bulletin. 72(9), 1319–1330 (1961).

[b8] Jakobsson M. . The international bathymetric chart of the Arctic Ocean (IBCAO) Version 3.0. J. Geophysical Research Letters. 39(12), doi: 10.1029/2012GL052219 (2012).

[b9] Geocap 7.1.1 for Windows http://www.geocap.no/ (2015).

[b10] Piskarev A. L., Andreeva I. A. & Gus’kova E. G. Paleomagnetic data on the sedimentation rate near the Mendeleev Rise (Arctic Ocean). J. Oceanology. 53(5), 620–629 (2013).

[b11] Elkina D. V., Pliocene-Quaternary sedimentation rates on the Mendeleev Ridge, the Arctic Ocean: Paleomagnetic studies. Open Transactions on Geosciences. 1(3), 12–214, doi: 10.15764/GEOS.2014.03002 (2014).

[b12] Kirschvink J. The least-squares line and plane and the analysis of palaeomagnetic data. Geophysical Journal International 62(3), 699–718 (1980).

[b13] Glebovsky V. Yu. . Formation of the Eurasia Basin in the Arctic Ocean as inferred from geohistorical analysis of the anomalous magnetic field. J. Geotectonics. 4, 21–42 (2006).

[b14] Owens P. N. . Fingerprinting and tracing the sources of soils and sediments: Earth and ocean science, geoarchaeological, forensic, and human health applications. J. Earth-Science Reviews. 162, 1–23 (2016).

[b15] Buchs D. M., Cukur D., Masago H. & Garbe-Schönberg D. Sediment flow routing during formation of forearc basins: constraints from integrated analysis of detrital pyroxenes and stratigraphy in the Kumano Basin, Japan. J. Earth and Planetary Science Letters 414, 164–175 (2015).

[b16] Gorbarenko S. A. . Magnetostratigraphy and tephrochronology of the Upper Quaternary sediments in the Okhotsk Sea: implication of terrigenous, volcanogenic and biogenic matter supply. Marine Geology. 183, 107–129 (2002).

[b17] Thiede J. and the Shipboard Scientific Party. Polarstern Arctis XVII/2. Cruise Report: AMORE 2001 (Arctic Mid-Ocean Ridge Expedition). Ber. Polarforsch. Meeresforsch. 421 (2002).

[b18] Jokat W. & Schmidt-Aursch M. C. Geophysical characteristics of the ultraslow spreading Gakkel Ridge, Arctic Ocean. Geophys. J. Int. 168, 983–998 (2007).

[b19] Snow J. E. & Edmonds H. N. Ultraslow-spreading ridges. Rapid paradigm changes. Oceanography. 20(1), 90–101 (2007).

[b20] Sohn R. A. . Explosive volcanism on the ultraslow-spreading Gakkel Ridge, Arctic Ocean. Nature. 453, 1236–1238 (2008). 18580949 10.1038/nature07075

[b21] Lane Ch. S., Chorn B. T. & Johnson T. C. Ash from the Toba supereruption in Lake Malawi shows no volcanic winter in East Africa at 75 ka. Proc Natl Acad Sci USA 110(33), E3048, doi: 10.1073/pnas.1301474110 (2013). 23630269 PMC3657767

[b22] Kim B. I. & Ivanova N. M. On the age of seismic units revealed on the Laptev Sea continental slope and adjacent part of the Eurasian Basin. Geological-geophysical features of the lithosphere in the Arctic Region. 3, 82–92 (VNIIOkeangeologia, St. Petersburg, 2000).

[b23] Piskarev A. L. Petrophysical models of the Earth crust in the Arctic Ocean. Transactions of VNIIOkeangeologia. 203, 134 (2004).

[b24] Gee J. S. & Kent D. V. Source of oceanic magnetic anomalies and the geomagnetic polarity time scale. Treatise on Geophysics Geomagnetism. 5, 455–507 (2007).

[b25] Zhamoida A. I. . Supplements to the Stratigraphic Code of Russia (ed. Zhamoida A. I.) 112 p. (VSEGEI, St. Petersburg, 2000).

[b26] Piskarev A. L. & Elkina D. V. Pliocene-Quaternary sedimentation rates on the Mendeleev Rise: Paleomagnetic studies. J. Carotazhnik. 5, 3–15 (2014).

[b27] Elkina D. Pliocene-Quaternary Sedimentation Rates of the Mendeleev Ridge: Paleomagnetic Studies. Master thesis: *Saint*-*Petersburg State University*; *University of Hamburg*. Saint Petersburg, Russia; Hamburg, 90 (2013).

